# A test of Generalized Bayesian dating: A new linguistic dating method

**DOI:** 10.1371/journal.pone.0236522

**Published:** 2020-08-12

**Authors:** Taraka Rama, Søren Wichmann

**Affiliations:** 1 Department of Linguistics, University of North Texas, Denton, Texas, United States of America; 2 Leiden University Centre for Linguistics, University of Leiden, Leiden, Netherlands; 3 Laboratory of Quantitative Linguistics, Kazan Federal University, Kazan, Russia; 4 Beijing Advanced Innovation Center for Language Resources, Beijing Language University, Beijing, China; Museum National d’Histoire Naturelle, FRANCE

## Abstract

In current practice, when dating the root of a Bayesian language phylogeny the researcher is required to supply some of the information beforehand, including a distribution of root ages and dates for some nodes serving as calibration points. In addition to the potential subjectivity that this leaves room for, the problem arises that for many of the language families of the world there are no available internal calibration points. Here we address the following questions: Can a new Bayesian framework which overcomes these problems be introduced and how well does it perform? The new framework that we present is *generalized* in the sense that no family-specific priors or calibration points are needed. We moreover introduce a way to overcome another potential source of subjectivity in Bayesian tree inference as commonly practiced, namely that of manual cognate identification; instead, we apply an automated approach. Dates are obtained by fitting a Gamma regression model to tree lengths and known time depths for 30 phylogenetically independent calibration points. This model is used to predict the time depths of both the root and the internal nodes for 116 language families, producing a total of 1,287 dates for families and subgroups. It turns out that results are similar to those of published Bayesian studies of individual language families. The performance of the method is compared to automated glottochronology, which is an update of the classical method of Swadesh drawing upon automated cognate recognition and a new formula for deriving a time depth from percentages of shared cognates. It is also compared to a third dating method, that of the Automated Similarity Judgment Program (ASJP). In terms of errors and correlations with known dates, ASJP works better than the new method and both work better than automated glottochronology.

## Introduction

The assignment of age to a proto-language has long been a desideratum in historical linguistics, and with [1] and in subsequent works by Swadesh a quantitative method was developed based on the hypothesis that the replacement of core lexical items is approximately constant over time. This method has been criticized extensively, mainly through examples showing that lexical replacement rates can vary dramatically [2]. Debates over glottochronology [3–5] cannot be regarded as settled since little is known statistically about the variability of lexical change across the world’s languages. We therefore include as part of this paper an extensive test of a modern version of glottochronology. As a matter of fact, a recent Bayesian phylogenetic study [6] found that a Turkic languages dataset supports a strict clock model, which assumes a constant rate of change, over the commonly used relaxed clock model, which allows for variable rates of change, suggesting that the assumptions of glottochronology can still be valid within the Bayesian phylogenetic dating framework. Holman and collaborators [7] introduced an alternative to glottochronology that used automatically measured string similarities rather than cognate counts as input to a linear formula for deriving dates. Another objective of the present paper is to also include this method in a performance comparison. Meanwhile, the related field of biology has seen application of Bayesian methods for inferring ages of species based on molecular data. In particular, there have been methodological developments in terms of increasing model complexity starting from a constant rate assumption to the development of models that handle varying rates across the branches [8, pp. 361-388]. The third and most important focus of this paper is to test whether Bayesian dating of languages can meaningfully be carried out in a framework which, like the ASJP method, is generalized in the sense that every language group is approached in the same way.

Early Bayesian linguistic phylogenetic studies of the Indo-European [9] and Austronesian [10] language families inferred dates using a combination of outgroups, rate smoothing methods, and calibration points associated with internal nodes. The next generation of Bayesian linguistic phylogenetic studies employed tip dating or node dating [11] techniques to jointly infer divergence times and phylogenies. These approaches have been applied to the Indo-European [12–14], Dravidian [15], and Sino-Tibetan [16, 17] language families. The success of Bayesian approaches as applied to language data has traditionally been dependent on the availability of information regarding an ancestral language (e.g., Old English), a language pertaining to a branch no longer represented by extant languages (e.g., Hittite), or an internal node which can be associated with archaeological (e.g., Iranian) or historical (e.g., Romance) evidence for the diversification of the corresponding group of languages.

### Vagaries of Bayesian dating

Bayesian methods for dating groups of related languages are popular, but the results are often debated, and the debates are rarely resolved—for instance, see [9, 12–14, 18] for debates concerning the Indo-European family. While the literature points to some degree of consensus on the fruitfulness of Bayesian approaches to the dating of language groups, the results of these approaches are less prone to consensus. The information used to construct dated phylogenies normally comes from laboriously assembled, manually curated cognate data, and researchers have relied on dates from archaeology or attestations of ancient languages as priors in the dating procedure—information which is only available in a minority of cases. Thus, only a small handful of language families have been dated through these methods in spite of the existence of 233 families [19] of spoken languages and an ever-present need for both individual information on each of the world’s human populations and systematic studies of the dynamics of human populations on a worldwide scale.

The choice of priors, differences in manual cognate judgments, and choices relating to calibration can all influence the age estimates produced by Bayesian phylogenetic inference methods leading to widely differing conclusions. In the case of the Indo-European family, the application of different tree priors (assumptions about the evolution of phylogenies, specifically ‘coalescent’ [20] vs. ‘birth-death’ [21]) on the same dataset produced root age estimates that differed by 2, 000 years [13]. Manually produced cognate sets have nearly always constituted the input data to the Bayesian approaches, but judgments about cognacy may vary among scholars and these judgments directly influence the structure and quality of the phylogeny inferred. For instance, correction of the lexical data [12] for both lexical errors and cognate judgments showed that the corrected datasets can lead to ages that are different—both younger and significantly different in terms of the Bayes Factor criterion [22]—from the original ages inferred based on the original dataset [14]. This result has been confirmed in [13], a paper that used a different set of tree priors but nevertheless reached similar conclusions as [12]. In the case of the Sino-Tibetan language family, a comparison of two recent papers [16, 17] shows that the choice of cognate sets, language sample, and calibration points produces root age estimates differing by 1500 years. Finally, different tree calibrations can yield different root ages, as illustrated by a case study of Uralic [23], where the calibrations used in a previous paper [24] were revised, yielding a 850 year (15+%) increase in the age estimate and a 3000 year widening of the credible interval (i.e., the 95% highest posterior density [HPD] interval).

### Research questions

For the vast majority of the world’s language families calibrating internal nodes based on archaeology and history will not be applicable, and even when such calibrations are possible, they are fraught with difficulties related to subjective judgments. In this paper, we are interested in looking at whether Bayesian dating of language groups can be brought on a more generalizable and objective footing. Specifically, we want to (1) infer ages for a language family with no calibration points; (2) replace the manual work on identifying cognates with an automated procedure. Through (1)–(2), Bayesian phylogenetic dating would be turned into a consistent and objective instrument, and, importantly, its results can now be evaluated across language groups since the input is no longer specific to the researcher choosing priors and calibration points and making cognacy decisions. The new approach, which we refer to as Generalized Bayesian Dating (GBD), would combine the strengths of the Bayesian and the ASJP approaches: like the Bayesian approach, it assumes an explicit model of lineage diversification, uses cognate sets and phonological information, handles across-site and among-branch variation, and produces estimates of uncertainty through posterior tree samples; like the ASJP approach, it can be applied for a language family even when there is no calibration information regarding some of the internal nodes in the tree.

## Materials and methods

### Materials

We perform all our experiments using Version 18 of the ASJP dataset, which is available online [25]. This dataset has word lists for more than 5,000 languages of the world, making it the largest online lexical database available in terms of language coverage. In order to develop and test a generalized method for dating language group divergence, a set of language groups for which there is information about when their shared proto-language was spoken is needed. Such a set of (fifty-two) calibration points is already available [7]. Dates for the various groups come from historical, archaeological or epigraphic evidence. We also use those dates here, but not all of them. Rather, we apply four selection criteria as described in the following paragraph.

For a tree-based approach to dating it is sensible to require at least three taxa since three taxa would constitute a rooted phylogeny. More specifically, (1) for a calibration point to be used we require that the pertinent data include at least three taxa carrying different ISO-639-3 codes. In general, more taxa make for better age estimates. Thus, the second of our criteria is to (2) choose a more inclusive group when the choice between a less or more inclusive group would lead to the same number of calibration points. Another criterion, (3) is to have as many calibration points as possible, but (4) without getting overlaps in terms of the doculects that they contain.

As regards the last criterion we differ from the authors of the ASJP study [7], who included among their calibration points some groups contained in larger groups. For instance, not only were Indo-European subgroups such as Brythonic, East Slavic, and Germanic included in their analysis, but also Indo-European (minus Anatolian and Tocharian), the wider family containing those groups. In the present study, in contrast, Indo-European was excluded as a calibration point. Out of the 52 calibration points, 8 points are excluded because they are not represented in our data by three or more doculects with different ISO 639-3 codes (Cham, Ga-Dangme, Goidelic, Sorbian, Northern Roglai Tsat, Ket-Yugh, Southwest Tungusic, Czech-Slovak); Khoe-Kwadi was taken out because the only member of one of the two major branches, Kwadi, is excluded for being extinct. A language group corresponding to Swahili could not be included since it is a subgroup of the larger Benue-Congo group, selected here because it has a greater number of languages than Swahili (23). By criteria (1-4) we arrived at the 30 core groups given in Table 1.

**Table 1:** Language groups representing calibration points, family membership, number of doculects in the data (N), and known age. The known age sometimes represent means of intervals given in the sources of the estimates.

Group	Family	N	Known Age	Group	Family	N	Known Age
Benue-Congo	Atlantic-Congo	148	6500	Mississippi Valley Siouan	Siouan	8	2475
Brythonic	Indo-European	3	1450	Mongolic	Mongolic	8	750
Chinese	Sino-Tibetan	16	2000	Ongamo-Maa	Nilotic	4	1150
Cholan	Mayan	5	1600	Oromo	Afro-Asiatic	5	460
Dardic	Indo-European	28	3550	Pama-Nyungan	Pama-Nyungan	68	4500
East Polynesian	Austronesian	18	1050	Romance	Indo-European	49	1729
East Slavic	Indo-European	4	760	Romani	Indo-European	26	650
English-Frisian	Indo-European	3	1550	Saami	Uralic	6	1750
Ethiopian Semitic	Afro-Asiatic	17	2450	Scandinavian	Indo-European	10	1100
Hmong-Mien	Hmong-Mien	38	2500	Southern Nilotic	Nilotic	14	2500
Inuit	Eskimo-Aleut	6	800	Southern Songhai	Songhay	5	550
Iranian	Indo-European	26	3900	Temotu	Austronesian	10	3200
Ma’anyan-Malagasy	Austronesian	44	1350	Tupi-Guarani	Tupian	12	1750
Malayo-Chamic	Austronesian	32	2400	Turkic	Turkic	55	2500
Maltese-Maghreb Arabic	Afro-Asiatic	3	910	Wakashan	Wakashan	6	2500

The present analyses were carried out using the 40-item Swadesh lists of the ASJP database [25]. Only lists that are at least 70% complete (containing 28 or more items) were used and no extinct languages or creoles were admitted. Following the Glottolog classification [19], we have aimed at maximizing the number of subgroups represented in the data while, for reasons of computational load, restricting the number of doculects for any given family to a maximum of 400. The choice of working with the short, 40-item word lists of the ASJP database [25] rather than longer word lists is a motivated one. We considered using publicly available datasets that have cognate sets for longer lists of meanings, including databases of Indo-European [12] (210 items), Bantu [26] (100 items), Austronesian [10] (210 items), and Pama-Nyungan languages [27] (200 items). However, these datasets are limited to a few language families, do not cover all calibration points listed in Table 1, and do not share the same set of meanings. Thus, for maximal coverage and a meaningful comparison across language groups, we only work with the ASJP database.

Raw data and code for selecting word lists and performing cognate identification is provided as separate folders in S1 File.

### Methods

#### Generalized Bayesian dating

*Automated cognate detection*. There is a rich literature on developing automated cognate detection methods [28, 29] for the purpose of detecting cognates and inferring phylogenetic trees [30, 31]. The automated cognate detection methods compute a similarity between two words based on hand-crafted phonetic similarity measures [32, 33], linear classifiers using word similarity scores [34, 35] or phoneme n-grams as features for training [36, 37] on hand-annotated training data, and neural networks [38]. Subsequently, the systems apply different clustering algorithms such as InfoMap [39] or UPGMA [40] to infer cognate clusters. Research on the performance of different clustering algorithms at the task of cognate detection [29, 41] shows that tuning the threshold can improve the results even for the simplest one, which is the average linkage clustering algorithm.

A recent paper [42] introduced a new clustering algorithm for cognate detection inspired by the Chinese Restaurant Process (CRP) [43], which does not require any threshold for forming cognate clusters. The CRP algorithm forms cognate clusters by linking a word to the cluster to which it has the highest similarity or to itself, where the self-similarity in a singleton cluster is penalized by multiplying it by a discount parameter *α*(> 0). The *α* parameter is tuned through a Metropolis-Hastings step for each meaning separately. Overall, the paper shows that the clusters formed by the CRP algorithm are of higher quality than the clusters using the Infomap algorithm, which works with a single threshold for all the meanings. The CRP algorithm has been evaluated on word lists belonging to the Austronesian, Austro-Asiatic, Pama-Nyungan, Indo-European, and Sino-Tibetan language families. Approaches [44] that infer trees based on an explicit model of sound change do not perform cognate detection at all and therefore cannot be applied to Swadesh list data but only to cognate data from etymological dictionaries; or else, they are not yet scalable for datasets consisting of hundreds of languages [45].

Recent research in computational historical linguistics [30, 46] has shown that the trees inferred using cognates inferred from automated methods are as good as those inferred from expert annotated cognate judgments. We believe that the next area for application of these cognate detection methods is in linguistic dating, because the dating process has traditionally been heavily dependent on manual cognate detection, which is time consuming, potentially biased, and not yet available for most of the world’s language families.

*Using both cognate and sound class data*. Bayesian phylogenetic studies typically use cognate classes to infer phylogenies, but it has been shown [46] that using both cognates and sound classes give better results for phylogenetic inference when drawing upon (an earlier version of) the ASJP database. We employ the automated cognate identification system described above [42] to assign cognate judgments to word lists. The choice of using such a system is supported by the results from two studies [30, 46], which show that automatically inferred cognates can yield high quality phylogenetic trees. In addition to the cognate characters, we operate with sound class characters, which are extracted as follows. Each word representing a given meaning is transformed into a set of characters representing the presence or absence of each of the sound classes found across words belonging to the given meaning. For instance, Swedish *hund* and English “dog” would yield a total of 7 characters, each representing a sound class in the set {o, u, d, g, h, n}.

*Bayesian phylogenetic inference*. All phylogenetic analyses were performed with MrBayes [47]. Cognate characters and sound class characters were treated as separate partitions and were subjected to a partition analysis. We used a birth-death model derived from the fossilized Birth-Death tree prior [48] with fossilization rate set to 0 since there are no extinct languages in the data. The birth-death model handles incomplete language sampling through a parameter *ρ* = *n*/*N*, where *n* is the number of languages in the sample and *N* is the total number of extant languages in the family. For families represented by more than 400 doculects we restricted the sample size to 400 doculects for reasons of processing time. The families in question and the number of selected vs. available doculects are: Austronesian (355/1254), Atlantic-Congo (265/1500), Afro-Asiatic (151/364), and Indo-European (205/462). In these cases the doculects were selected so as to maximize the phylogenetic diversity. Specifically, we applied the following procedure: first we selected the best documented doculect (the one with the largest number of words or a random one in case of ties) from the subgroups at the deepest level of the Glottolog classification and checked the size of the sample. If it was still greater than 400 we went up one level, again selecting one doculect per subgroup. This continued until the sample was below 400. Moreover, we corrected for extant taxa sampling bias through diversified sampling correction [49] implemented in [48]. We used the number of extant doculects in the ASJP database as representing the number of extant taxa, *N*, and fixed *n* (the number of sampled taxa) to be the number of doculects in the sample. In the case of families of size less than 400, we included all the doculects and assumed *ρ* = 1. Setting *N* to the number of extant doculects in the database and using *ρ* = 1 for smaller families are both approximations. But they are motivated by the facts that the database contains 7655 doculects and the number of languages in the world is 7570 by a widely used estimate [50], so it would be a fair approximation on average. Moreover, a consistent and objective criterion for counting languages vs. dialects [51] has not yet been applied in any language catalogue, so setting these parameters will in any case involve approximations. The birth-death prior used in this paper is conditioned on the root height, which has to be specified beforehand. Since the tree is uncalibrated, the root height is drawn from a Gamma distribution with rate and shape parameters set to 1.

*Lexical substitution model*. We model variation across sites for each partition separately. The Bayesian partition analysis assumes that each partition has a different rate (drawn from a symmetric Dirichlet distribution), which enters the calculation of the likelihood for a site through its multiplication with the branch lengths in the tree. In this paper, we employ a continuous time Markov chain process model for binary states (binCTMC) with site rates drawn from a four category discrete gamma distribution (binCTMC+Γ model) in all phylogenetic analyses, correcting for all-absence sites [52]. We prefer binCTMC model over other alternative substitution models such as covarion and stochastic Dollo for the following reasons. The binCTMC model is a single parameter model whereas the covarion model has three parameters, with binCTMC being a special case of the covarion model. In the stochastic Dollo model, a cognate can be gained only once, which prohibits regaining of a cognate through a process such as internal borrowing. The suitability of these substitution models has been shown to be variable, with binCTMC being the best suited for Indo-European [12] and covarion the best suited for Sino-Tibetan [17], and a comparative study of different substitution models on four different language families, including Indo-European [53], suggest that there is no clear winner between binCTMC and covarion models. Thus, we selected the binCTMC+Γ model for its simplicity and competitiveness against the more complex covarion model.

*Branch lengths*. A branch length in a time tree is the product of calendar time units and clock rate. The clock rate, *c*, measures the average number of substitutions that can occur per year per site and is assigned a prior. If the clock rate is assumed to be constant for the whole tree the model it is a strict clock rate. It is normal to relax this assumption since branches can have varying rates of evolution. The branch lengths, then, are further multiplied by a factor drawn from a distribution which is parameterized in terms of the original branch length. There are multiple relaxed clock rate models [54] employing different assumptions for deriving the multiplicative factor. In this paper, we use an Independent Gamma Rate [54] relaxed clock model (IGR) where each branch’s rate comes from different Gamma distributions whose mean is 1.0 and variance is proportional to the inverse of the branch length. We fix the clock rate to 1 but allow the branches to have different rates drawn from the IGR model, thereby allowing the branch rates to deviate from the strict clock assumption.

In this paper, we assume that the joint prior probability of the branch lengths under a birth-death process is conditioned on the tree height which in turn is drawn from a Gamma distribution with mean and variance set to 1. Does the tree prior assumption have any bearing on distribution of tree lengths? While we could not find any research showing a relation between tree length (in a birth-death process) and a known probability distribution, there are results [55] showing that the tree length in a Yule process (pure birth process) conditioned on tree height is dependent on a Gamma distribution, whereas the same Yule process, if conditioned on the number of extant languages, follows a Gamma distribution. (The Yule process, incidentally, is not a realistic model for languages since it is well known that languages may die out.) We acknowledge that the statistical properties of tree length in a birth-death tree conditioned on tree height is not yet known.

*Topological constraints*. As in most previous Bayesian phylogenetic studies we employ topological constraints, but unlike our predecessors we introduce as many constraints as possible since we are not concerned with language classification but only with the dating of recognized subgroups. A recent study [46] using the maximum likelihood phylogenetic software RAxML [56] showed that trees inferred from word lists in the ASJP database largely agree with the Glottolog [19] classification in terms of quartet distances [57]. We therefore find it justifiable to not take the extra step of inferring all nodes in the phylogenies but rather to fix most of the nodes of the tree based on the Glottolog classification. The limit on constrained nodes are determined by the data: as mentioned earlier, we require that each constrained node should suspend at least three doculects. All in all we extracted 1171 topological constraints from Glottolog.

*Monte-Carlo Markov chain settings*. For each family, we did two independent runs and sampled parameters by running one cold and two hot chains in parallel. The hot chains allow the MCMC chain to explore the parameter landscape efficiently by moving across the peaks and not get stuck in a local optimum. We ran the chains for different numbers of generations depending on the number, *N*, of doculects in a family: 10^7^ for *N* < 100, 3 × 10^7^ for 100 < *N* < 200 and 6 × 10^7^ for *N* > 200. The chain was sampled at every 1000th generation in order to reduce auto-correlation. We assessed the convergence of branch lengths using the Potential Scale Reduction Factor [58], whose value should approach 1 across independent runs should the runs converge. The independence between the samples for parameters such as tree height, birth-death rates, and the IGR variance parameter is assessed using Estimated Sample Size, which is normally required to be at least 100 for all our parameters [58]. The rate variation across sites is modeled using a discrete Gamma distribution with four rate categories [59].

The Nexus files and MrBayes command files are provided in the folder Nexus_MrBayes folder in S1 File. The output of MrBayes is available for download here: https://figshare.com/s/a2ba6dbb656b6cb0b9bb.

*Gamma regression*. Gamma regression is a special case in the Generalized Linear Model (GLM) framework [60], where the response variable follows a Gamma distribution. An exploratory data analysis showed that a log-log plot of known age vs. median tree length (MTL) is linear. We choose Gamma distributed ages over normally distributed ages for the following reasons: (1) The age of a language group is always positive, (2) The assumption of constant variance is not realistic because there is a difference in the variance in the ages of the language families, (3) A linear regression model on a log-log scale implies that the response variable has constant variance and is not on the original scale. These problems can be solved with a Gamma distribution with *ν* and λ as shape and rate parameters whose mean (*μ* = *ν*/λ) and variance are *μ*^2^/*ν*.

The Gamma distribution has positive support, non-constant variance, and removes the need for logarithmic transformation. The GLM framework features a smooth, invertible function that links the response variable with the predictor, which is a log function here. The parameters (coefficients: *β*_0_ and *β*_1_, and the shape parameter *ν*) can be estimated using an MCMC sampler in the Bayesian framework. The priors for *β*_0_ and *β*_1_ are uniform distributions in the range of −100 to 100. The *ν* parameter is always positive and the prior is a uniform distribution ranging from 0 to 100. The posterior distribution of the parameters are obtained by running the MCMC chain for 10^6^ generations and sampling at every 100th generation to reduce auto-correlation. The initial 25% of the generations are discarded as burn-in and the independence of the samples is assessed using the Estimated Sample Size (> 1000) for all the parameters in our experiments. All MCMC samplers are implemented using the RevBayes programming language [61].

In the following, we evaluate the choice of the Gamma regression model against the linear regression model using AICM (Akaike’s Information Criterion through MCMC [62]), where the best model has the lowest AICM. The prior for variance (in the linear regression model) is exponentially distributed with mean 1. The AICM values are estimated using the Tracer software [63] from the post-burnin (25% discarded) samples for the four models. The model with the lowest AICM (shown in bold) is the best fit for the data. The first three models assume that the age of a family follows a Gamma distribution whereas the last line shows a model where a known age is distributed normally. The null model for Gamma regression has a poor fit whereas the regression model with normally distributed ages shows the worst fit to the data. The results of the model evaluation are presented in Table 2. The AICM values show that Gamma distributed age model is better than a normally distributed age model.

**Table 2:** AICM values for different models. The first four lines show Gamma regression models using different predictors. The last line shows a linear model with logarithm of MTL as predictor.

Model	AICM
Age ∼ Intercept (Null model)	507.521
Age ∼ log(MTL)	**490.108**
Age ∼ MTL	493.199
Age ∼ log(MTL) (Normally distributed Age)	854.408

*Inferring ages*. The MCMC procedure yielded 7500 Gamma regression parameters sets. We employed each set of parameters to compute the age distributions using the median tree length for the topological constraint. Subsequently we report the median and 95% HPD intervals for the ages from the computed ages.

Although the sum of the branch lengths is used to compute the tree length of a language group, the relaxed clock setting (Independent Gamma Rates; IGR) applied in the tree inference procedure works with a slightly modified branch length called effective branch length, which is obtained by multiplying a branch length with a rate drawn from a Gamma distribution. The effective branch length is used to compute the likelihood of the tree using the pruning algorithm. We experimented with regard to the relation between the median effective branch lengths and the median branch lengths through a scatterplot for all the topological constraints. A linear trendline between both types of branch lengths shows a slope of 1.031, an intercept of −0.004 and *R*^2^ = 0.94. We therefore use the unmodified branch lengths to compute the tree lengths.

The choice of median tree length as opposed to mean tree length is motivated by the fact that median tree length corresponds to the length of one actual tree, whereas the mean is a more abstract number, being computed from the sample of tree lengths. The two are in any case highly correlated for the 116 language families (*R*^2^ = 0.997). We always report the median statistic for all ages and branch lengths.

The exponential function’s parameters, *a* = 1843 and *b* = 0.35, follow from the median estimates of *β*_0_, *β*_1_ which are 7.52 and 0.35. The formula is *t* = 1843 × *MTL*^0.35^. When MTL tends towards 0, as is the case of close dialects, the age of the most recent common ancestor also tends towards 0.

In earlier exploratory experiments (not reported in detail here), *R*^2^ is almost zero when tree height is used as a predictor for the 30 groups rather than tree length. So, we adopted tree length which is clearly to be preferred over tree height.

The Means, medians, standard deviations and 95% HPD intervals for these dates are given in S1 Table. In addition, consensus trees for all language families annotated with inferred dates are provided in Consensus_Trees_Ages folder in S2 File.

#### Non-Bayesian approaches

We tested two non-Bayesian approaches: the published version of ASJP chronology [7] and a version of traditional glottochronology [64] which is modernized so as to draw upon automated rather than manual cognate counts and where the calibration of cognate percentages and age comes from the same 30 calibration points used in our Bayesian dating experiment rather than a few language pairs from the literature [65]. In both cases, for each group, a flat classification structure consisting of the same major subgroups assumed in the paper introducing ASJP chronology [7] was defined. These subgroups are usually the same as in Ethnologue [50], but in a few cases a date is found in the literature for a language group not considered a subgroup in that classification although it is still compatible with it. In such cases the classification followed here will differ from [50], but it will be the same as in [7]. The classification divides doculects into sets for the purpose of computing an average cognate count or average twice-modified Levenshtein distance (LDND) between all pairs of doculects whose members belong to different sets. When there is no internal classification of a group, such as in the case of Chinese, the sets are defined following the ISO-639-3 standard. Thus, for instance, for Wakashan an average is found for all pairs where one doculect pertains to Northern and another one pertains to Southern Wakashan. For Chinese, however, the sets are defined as [cdo], [cmn], [cpx], etc., and the average is found for all doculects pertaining to different sets of this kind.

*ASJP chronology*. The procedure for ASJP chronology is described in [7] and will only be briefly summarized here. The basic input to the method is the twice-modified Levenshtein distance called LDND (Levenshtein Distance Normalized Divided) used in many papers of the ASJP project and extensively discussed in [66]. This is turned into a similarity, LSND (Levenshtein Similarity Normalized Divided), by subtracting LDND from 100%. LSND can be abbreviated *s*. The LSND (or *s*) can be directly converted into an age estimate through a formula *t* = (*log*(*s*) − *log*(*s*_0_))/2*log*(*r*), where *s*_0_ is similarity at *t* = 0, *r* is a retention rate, and *s* is the measured similarity. Here we use the values *s*_0_ = 92% and *r* = 0.72 that were established in [7] using 52 calibration points. Thus, our application of ASJP chronology is strictly orthodox. We use the original, published software for computing dates—Holman’s asjp62c.exe at https://asjp.clld.org/software (alternatively our own R software at https://github.com/Sokiwi/InteractiveASJP01 could be used). Holman’s program requires standard ASJP-style input files and looks for the Ethnologue classification, which is located between | and @ in the first line of metadata for each language. Since we use the Glottolog classification here, we have moved that to between | and @. Moreover, the program takes as part of its input the level of a given classification for which one wants to produce dates. This level can be set to 1 for the top level provided that the classification string starts at the level one is interested in. This is the practice followed for files given as input for asjp62c.exe. For the sake of replicability these input files are provided in the S3 File along with the outputs. The dates extracted from the outputs are listed in Table 6.

*Automated glottochronology*. Automated glottochronology is highly similar to traditional glottochronology except that the cognate counts are based on automatically inferred cognates. Moreover, while we infer a constant rate of change through a linear model calibration, as did [65], we allow the intercept to not be equal to 0, taking the insight from [7] that languages have internal variation such that lexical similarity at 0 years of separation is not necessarily 100%. Finally, we do not adopt a retention rate from the glottochronological literature, something which would probably disadvantage the method. Instead, for each of the 30 language groups we fit a linear model in a leave-one-out fashion, basing the linear model on the remaining 29 calibration points. The cognate proportions for each language group represent the number of cases where two doculects have the same cognate class for a given meaning divided by the number of meanings for which both doculects have attested words. The final score for a language group represents an average over pairs whose members belong to the different Glottolog subgroups. All relevant files and an R-script executing each step towards computing the final dates are included in S4 File.

The purpose of the exercise *is not to revive glottochronology* but to evaluate the method and derive a baseline for evaluating the performance of the other dating methods.

## Results

### GBD validation

What is the quality of the predictions made by GBD? In the following subsection we compare the predictions with those of other methods. In the present subsection we answer the question through leave-one-out validation. We exclude each calibration point successively and then fit a Bayesian Gamma regression model based on the rest of the 29 calibration points. Then we use the inferred parameters from the 29 points based model to predict the age ranges for the excluded calibration point. Overall, we fit 30 models and perform predictions based on each model. The predictions of these experiments are given in Table 3. We report the difference between the median predicted age and the known age as well as the 95% HPD interval of the age predictions.

**Table 3:** Difference between the predictions from leave-one-out validation and the known ages. We report the median age difference and 95% HPD interval of the prediction.

Subgroup	Difference and Range	Subgroup	Difference and Range
Benue-Congo	2414 [6182-2548]	Mississippi Valley Siouan	736 [2033-1450]
Brythonic	390 [1377-778]	Mongolic	-2037 [3442-2214]
Chinese	101 [2238-1576]	Ongamo-Maa	192 [1315-690]
Cholan	371 [1527-960]	Oromo	-776 [1564-972]
Dardic	1313 [2713-1862]	Pama-Nyungan	695 [5199-2469]
East Polynesian	-530 [1894-1280]	Romance	-926 [3247-2113]
East Slavic	-348 [1494-874]	Romani	-1218 [2166-1564]
English-Frisian	637 [1215-627]	Saami	108 [1915-1350]
Ethiopian Semitic	735 [2010-1412]	Scandinavian	-375 [1747-1181]
Hmong-Mien	-924 [4558-2365]	Southern Nilotic	524 [2354-1688]
Inuit	-952 [2069-1454]	Southern Songhai	-917 [1763-1216]
Iranian	1340 [3129-2049]	Temotu	1595 [1928-1359]
Ma’anyan-Malagasy	-686 [2399-1715]	Tupi-Guarani	-136 [2214-1595]
Malayo-Chamic	259 [2605-1784]	Turkic	-806 [4452-2431]
Maltese-Maghreb Arabic	-92 [1348-695]	Wakashan	-262 [3428-2151]

The largest errors of the model are for Benue-Congo and Mongolic, where Benue-Congo is the oldest point and Mongolic is a younger data point. For several points, such as Dardic, Iranian, and Mongolic, the known ages are outside the predicted age range. The mean absolute error and the root mean square error are 770 years and 936 years respectively.

Since tree length is highly dependent on the number of tips in the tree, which are in turn a predictor of the age of the root, it is legitimate to ask whether the method is actually using the lexical data and is not just predicting the age of the language family based on its number of extant descendants. In order to investigate this we applied the same leave-one-out procedure using the logarithm of the number of doculects in the sample as a predictor. That produces a mean absolute error is 822 years and a root mean squared error of 1012 years. Since the error is considerably larger using the number of doculects than using MTL as a predictor we can conclude that MTL is not simply a function of the number of doculects and that using the latter in a dating procedure is not going to be productive.

### Comparison against other methods

Using the GBD method we obtained 116 dates for families or around 10 times as many dates for families including subgroups. These are provided for the record in S1 Table. Here we concentrate on the evaluation of the results and compare them to the results for ASJP dating and glottochronology for the 30 calibration groups.

Table 4 shows mean absolute error (absolute difference between an inferred and known date), root mean square error, and Pearson correlation for the results for GBD, ASJP, and glottochronology shown in Table 5. By any of these measures, ASJP shows better performance than GBD and GBD better performance than glottochronology. None of the methods’ differences, whether mean absolute error or root mean square error are used, are not significant by a Wilcoxon signed rank test, however.

**Table 4:** Comparison between GBD, ASJP, and glottochronology based on different evaluation measures.

Measures	GBD	ASJP	Glottochronology
Mean absolute error	695	606	728
Root mean square error	858	823	1039
Pearson’s *r*	0.76	0.78	0.61

**Table 5:** Language groups representing calibration points, known ages, and inferred ages using GBD, ASJP, and glottochronology. (‘M.V’ in M. V. Siouan stands for Mississippi Valley).

Group	Known Age	GBD	ASJP	Glot.chron.	Group	Known Age	GBD	ASJP	Glot.chron.
Benue-Congo	6500	4496	4781	2965	M. V. Siouan	2475	1760	1725	2404
Brythonic	1450	1085	1144	973	Mongolic	750	2683	2002	1684
Chinese	2000	1910	3066	2251	Ongamo-Maa	1150	977	1083	1678
Cholan	1600	1246	1142	1599	Oromo	460	1174	2086	2606
Dardic	3550	2276	1917	1984	Pama-Nyungan	4500	3905	4470	3653
East Polynesian	1050	1556	985	1070	Romance	1729	2608	1672	1208
East Slavic	760	1092	1124	1080	Romani	650	1823	614	919
English-Frisian	1550	982	1826	996	Saami	1750	1638	1532	1277
Ethiopian Semitic	2450	1717	2235	2587	Scandinavian	1100	1453	1172	954
Hmong-Mien	2500	3326	3957	3473	Southern Nilotic	2500	2007	2911	3273
Inuit	800	1710	945	1328	Southern Songhai	550	1437	594	859
Iranian	3900	2651	2344	2136	Temotu	3200	1670	3762	3405
Ma’anyan-Malagasy	1350	2016	2096	2300	Tupi-Guarani	1750	1879	1830	1979
Malayo-Chamic	2400	2162	2036	1714	Turkic	2500	3206	3414	2210
Maltese-Maghreb Arabic	910	999	1477	1689	Wakashan	2500	2760	3626	4070

## Discussion

### Comparison between GBD results and published Bayesian dates

A comparison (Table 6) between the root ages as inferred in prominently published Bayesian dating analyses for five language families and our Generalized Bayesian dating method shows good agreement. The predicted median root age (4800 years) of the core Indo-European group (i.e., excluding Tocharian and Anatolian) is very close to the age (∼4800) inferred in the Bayesian study [12], which dated the Indo-European tree through the input of tip dates from 17 non-extant languages. In the case of Austronesian, our method predicts a median root age of 5600 years whereas the Bayesian study [10] employing different internal calibration points inferred an only slightly younger mean age of 5300 years. For Sino-Tibetan our approach infers a median root age of 5100, which is not far from the 5900 years inferred in one recent study [17], although further from the 7100 years inferred in another [16]. The median root age of 3919 years found for Pama-Nyungan is younger than the published date of 5671 years but close to the age range of 4000–5000 years given by experts based on archaeological and linguistic correlations [7]. Finally, our age for Dravidian is within the range given in the publication for that family [15], even if close to the margin.

**Table 6:** Comparisons between published Bayesian dates for different language groups and our results. We show the median and the 95% HPD intervals for each language family. The median and HPD intervals are obtained by using the parameter distribution of the Gamma regression from the formula shown in Methods section.

Language Family	Generalized Bayesian Dating	Published Bayesian Dates
Indo-European	4826 [7213-3074]	4818 [5528-4123] [12]
Austronesian	5647 [8831-3314]	5230 [5800-4750] [10]
Sino-Tibetan	5111 [7799-3199]	5900 [7800-4200] [17]
		7184 [9568-5093] [16]
Pama-Nyungan	3905 [5403-2676]	5671 [6966-4455] [27]
Dravidian	3293 [4285-2375]	4500 [6500-3000] [15]

Typically, the lower bounds of the 95% HPD intervals inferred by the Generalized Bayesian method are younger than those of Bayesian dates we list in Table 6. This is due to the fact that the oldest calibration point’s upper bound acts as a lower bound for the root age in the traditional Bayesian approaches, whereas we do not operate with such internal calibration points. For instance, in the Dravidian study [15], an age of 2250 years corresponding to Old Tamil inscriptions is employed as an internal calibration point, which forces the inferred family age to be at least as old as this internal calibration point.

### Does GBD have a potential to outperform ASJP chronology?

It is prudent to inspect invited critical comments on the ASJP paper [7] to look at the extent to which they carry over the present approach. More than one critic was dissatisfied with the classification used, which is alleviated in the present paper because of the high-quality Glottolog classification which has since become available. ASJP dates for Mixe-Zoquean, Mayan, Austronesian, and Uralic were criticized as being too young, while the date of Insular Celtic was said to be exaggerated (cf. comments by Adelaar, Blust, and Nichols). All these dates have shifted in the right directions in the present paper. For some ASJP dates it happens that a mother node is younger than a daughter node. This is no surprise given the uncertainty of the estimates, but is still awkward, as noted by Campbell and Adelaar. In the present, tree-based approach this phenomenon does not occur. As regards methodological comments, we avoid using calibration points with overlapping languages, as advised by Adelaar, and we incorporate quantification of error as advised by Blust. Some improvements still called for in the set of comments would be the improvement of dates for Afro-Asiatic and Semitic, which are probably too young in the ASJP and in the present one as well (cf. comments by Nichols). This might be achieved by the development of new methods to include data from the equivalent of biological fossils—extinct languages. Finally, as advocated by Embleton, longer word lists are a desideratum. While the ASJP approach, which makes use of a single, aggregated distance measure, might actually not benefit appreciably from longer word lists, it has been shown that the number of cognate classes required for optimal performance of Bayesian classification is directly proportional to the number of languages classified [67]. It is likely, then, that our Bayesian age estimates would also improve with additional lexical data. Fortunately, word lists longer than 40 items are presently being compiled by research groups working on languages in several different parts of the world, so it is likely that the present study can be replicated, expanded, and significantly refined within around a decade from now. Meanwhile, we leave the question contained in the title of this subsection open, but we believe that, as far as a generalized approach to the world-wide dating of language groups is concerned, the method applied and the data drawn upon are not easily paralleled.

### Shortcomings of GBD

Both the leave-one-out errors reported in Table 3, the errors from comparison against other methods in Table 5, and the age ranges given in Table 6 (complete list given in S1 Table) are quite large. We will first look at previous Bayesian studies and attempt to determine whether the kind of error is unique to GBD or is expected from Bayesian dating methods in general. To the best of our knowledge, only the study of [12] examines the systematic effect that each calibration point has on the Bayesian phylogenetic analysis. The study of [12] uses calibration points and lexical cognate data from eight ancient languages (cf. Table 7) to perform Bayesian dating analysis. The authors analyze the effect of leaving out a calibration point on the phylogenetic analysis through a leave-one-out analysis and allow the Bayesian phylogenetic program to infer the age of the ancient language. We provide the inferred dates from the paper’s supplementary material along with the known age of a language. The table shows that the median ages for five of the eight languages do not fall within the known age ranges. Moreover, the authors also show that Vedic Sanskrit, the oldest calibration point, improves the model fitness 20 times *strongly* [22] in terms of Bayes Factor. The main conclusion from this analysis is that a temporally deep calibration point is important for improving model fitness and the errors can be quite large even in the case of a language family with well-attested language dates.

**Table 7:** Age ranges for some ancient languages inferred in [12] through a leave-one-out analysis. Calibration points whose inferred median age does not fall within the known age range is indicated by a †. The third column shows how the root age ranges are affected by exclusion of an ancient language point.

Language	Known age range	Inferred age range	Inferred Root age range
Old Irish †	1300–1100	1603 [2363–988]	6120 [7390–5070]
Latin†	2200–2100	1797 [2457–1270]	5770 [6990–4780]
Old West Norse	850–750	839 [1200–506]	5930 [7270–4920]
Old English†	1050–950	1093 [1473–760]	6090 [7410–5040]
Old High German†	1100–1000	944 [1275–635]	5970 [7230–4970]
Ancient Greek†	2500–2400	1900 [2976–939]	5910 [7270–4850]
Classical Armenian	1600–1300	1544 [2291–835]	5970 [7360–4890]
Vedic Sanskrit	3500–3000	3150 [3848–2454]	5930 [7350–4590]

The root age ranges from previously published Bayesian studies using calibration points vary between 1000 years and 4500 years (cf. Table 6). It has to be noted that none of the inferred root ages is evaluated since we do not know the exact period during which the root languages actually split. We only conclude that the age ranges inferred by GBD are not worse than the published dates and more studies are urgently required to determine the shortcomings of the dating methods [68].

## Conclusion

In this paper we have introduced a new method called Generalized Bayesian Dating (GBD) for inferring dates of language groups from lexical and phonological data. It was tested against ASJP chronology and glottochronology. Both in terms of mean absolute error with respect to 30 known dates, root mean squared error, and correlations between those dates and the inferred ones, the rank of performance is such that ASJP works better than GBD, which works better than glottochronology. The results suggest that glottochronology is not worth pursuing further. As for the two best performing methods, it should be taken into account when evaluating them comparatively that GBD has certain *potential* advantages over ASJP, including a greater potential for future improvements using more extensive data.

While the results of any of the three methods are not as good as we would like them to be, there are similarly large error bars associated with Bayesian dates even when trees are supplied with internal calibration points. Thus, it is generally true that dating in historical linguistics is a tough problem, and in order to progress in this field it is better to expose the problems than to ignore them. Moreover, it is important to develop standards of comparison in order to be able to test different methods. This is the first paper that has attempted a comparative test of different methods using a large dataset, a single classification, and no prior assumptions specific to different language groups. If nothing else, we hope that the present work may inspire a new culture of scientific rigor in replicability and performance testing in the area of linguistic dating.

## Supporting information

S1 File Input generation for GBD: Code, cognate datasets and nexus files. Python code for extracting data from ASJP word lists, cognate identification, and conversion to nexus files. Word lists annotated for cognacy for all families. Nexus files and MrBayes command files for all families.
(ZIP)

S2 File Gamma regression model fitting and dates prediction. Python code for extracting median tree lengths for each constraint, generation of consensus trees, RevBayes code for performing Gamma regression and linear regression, and Python code for predicting ages. Age annotated consensus tree files for all the families.
(ZIP)

S3 File Input and output files for ASJP dating method. The word lists for 30 calibration points required for running the asjp62c program. The dates for each calibration point are also supplied along.
(ZIP)

S4 File Glottochronology data and output. Files, including an R script, for producing glottochronology dates from the output of the automated cognate detection procedure.
(ZIP)

S1 Table GBD age predictions. Excel file containing GBD dates for all the 116 families and 1171 constrained subgroups.
(XLSX)
