# Supplementary material for: A test of Generalized Bayesian dating: A new linguistic dating method
Source: PLoS One. 2020 Aug 12;15(8):e0236522. doi: 10.1371/journal.pone.0236522 (PMC7423060; doi:10.1371/journal.pone.0236522)
Supplement: S3 File — The word lists for 30 calibration points required for running the asjp62c program. The dates for each calibration point are also supplied along. (ZIP) [file pone.0236522.s003.zip › S3 Supporting Information/Instructions-asjp62c.pdf]

Eric W. Holman

Program for calculating ASJP dates (version 1.1) [2011]

Instructions

The program described here calculates ASJP dates for language families based on a similarity score 1 - LDND, where LDND is defined by Bakker et al. (2009). The procedure for calculating dates is described in Holman et al. (forthc.), the paper which this program accompanies.

The program uses a slightly modified regular ASJP datafile as input (downloadable from http://email.eva.mpg.de/~wichmann/languages.htm; cf. the instructions to Holman 2011 for a description of the format). The modification consists in adding three numbers to the first line, such that it takes the following form:

```
      2    24  1700     1    92    72
```

The 1 in col. 24 is the level in the Ethnologue classification (Paul 2009), which is cited within the curly brackets after | right after the language designations heading each wordlist. The program has to be run separately for each level. The 92 in cols. 29-30 is the value of s0 and the 72 in cols 35-36 is the value of r, both without decimal points. The values can be changed should a different calibration be used.

To run the program, use the MS-DOS command prompt, and type a command of the form asjp62 < input > output and then press Enter.

References

Bakker, Dik, André Müller, Viveka Velupillai, Søren Wichmann, Cecil H. Brown, Pamela Brown, Dmitry Egorov, Robert Mailhammer, Anthony Grant, and Eric W. Holman. 2009. Adding typology to lexicostatistics: a combined approach to language classification. *Linguistic Typology* 13.167-179.

Holman, Eric W. 2011. Programs for calculating ASJP distance matrices (version 2.1). <http://email.eva.mpg.de/~wichmann/software.htm>

Holman, Eric W., Cecil H. Brown, Søren Wichmann, André Müller, Viveka Velupillai, Harald Hammarström, Sebastian Sauppe, Hagen Jung, Dik Bakker, Pamela Brown, Oleg Belyaev, Matthias Urban, Robert Mailhammer, Johann-Mattis List, and Dmitry Egorov. Forthc. Automated dating of the world's language families based on lexical similarity. *Current Anthropology*.

Lewis, M. Paul (ed.). 2009. *Ethnologue*. 16th Edition. Dallas: SIL International. (www.ethnologue.com)
